# Expression of Two Basic mRNA Biomarkers in Peripheral Blood of Patients with Non-Small Cell Lung Cancer Detected by Real-Time RT-PCR, Individually and Simultaneously

**DOI:** 10.6091/ibj.1397.2014

**Published:** 2015-01

**Authors:** Shirin Karimi, Abdolreza Mohamadnia, Seyed Alireza Nadji, Reza Yadegarazari, Adnan Khosravi, Naghmeh Bahrami, Massoud Saidijam

**Affiliations:** 1 *Mycobacteriology Research Center, National Research Institute of Tuberculosis and Lung Diseases (NRITLD), Shahid Beheshti University of Medical Sciences, Tehran, Iran; *; 2 *Research Center for Molecular Medicine, Hamadan University of Medical Sciences, Hamadan, Iran;*; 3 *Dept. of Genetics and Molecular Medicine, Hamadan University of Medical Sciences, Hamadan, Iran; *; 4 *Virology Research Center, National Research Institute of Tuberculosis and Lung Diseases (NRITLD), Shahid Beheshti University of Medical Sciences, Tehran, Iran;*; 5 *Tobacco Prevention and Control Research Center, National Research Institute of Tuberculosis and Lung Diseases (NRITLD), Shahid Beheshti University of Medical Sciences, Tehran, Iran; *; 6 *Iranian Tissue Bank and Research Center Tehran University of Medical Sciences, Tehran;*; 7 *Dental Biomaterial Dept. of Dentistry, Tehran University of Medical Sciences, Tehran, Iran*

**Keywords:** Lung neoplasms, RNA, Carcinoembryonic antigen, Sensitivity and specificity

## Abstract

**Introduction: **Although extensive research has been conducted on lung cancer markers, a singular clinically applicable marker has not been found yet. The objective of this study was to evaluate the sensitivity and the specificity of carcinoembryonic antigen (CEA) mRNA and lung-specific X protein (LUNX) mRNA biomarkers in peripheral blood to detect lung cancer individually and simultaneously. **Methods: **Thirty patients affected by lung cancer and 30 healthy individuals were studied in this research. Three vials of cDNA were made from each sample after taking peripheral blood samples and extracting total RNA. Each sample was examined by the real-time RT-PCR technique. The result from each vial was then compared with the sensitivity of overall marker. **Results: **The CEA mRNA was positive in 24 out of 30 lung cancer patients. Hence, its sensitivity was determined at 80%, differing significantly from that observed in healthy individuals, where 11 positive cases were seen. The overall sensitivity of this marker was significantly associated with positivity in vials 2 and 3 but not in vial 1. The LUNX mRNA was positive in 21 out of 30 patients, indicating 70% sensitivity. This finding significantly differed from that in healthy individuals. The overall sensitivity of this marker was significantly associated with positivity in vials 1 and 3, but not in vial 2. In 93.3% of the patients, at least one positive marker was observed. **Conclusion: **The mentioned mRNA could be suggested as sensitive and specific markers in peripheral blood for primary diagnosis of lung cancer.

## INTRODUCTION

Lung cancer is the most common cancer affecting men across the world, and over 80% of those affected die within 5 years of diagnosis. [1]. The five-year prognosis of lung cancer is only 13% [2]. The risk of acquiring lung cancer is approximately 8% in men and 6% in women [3]. Epithelial lung cancers have four main cellular classifications: small cell lung carcinoma and also adenocarcinoma, squamous cell carcinoma, and large cell carcinoma, which are known as non-small cell lung carcinoma. These four types account for 90% of epithelial lung cancers [4, 5]. Tumor markers are considered as important tools for acquiring information regarding cancers, usually at a lower cost of time and money. These markers in body fluids are created either by the tumors or by contact with cancerous cells and may help in diagnosis, prevention, estimation of tumor size and determination of early cancer recurrence. They may also be beneficial for the selection and timing of treatment [6]. 

CEA (carcinoembryonic antigen) is a glycoprotein that is produced only in embryonic liver and is no longer produced afterwards [7]. Its reduction during treatment indicates cancer cell growth control [8]. The serum level of CEA mRNA has been studied in other cancers as well. The sensitivity of this marker is roughly 20% and 21% in gastric and breast cancer, respectively [9, 10], and it is higher in lung cancer [11, 12]. Thus, research on this marker has been more or less focused on prognosis and treatment [13, 14]. Therefore, CEA mRNA measurement in peripheral blood of lung cancer patients can be considered as an important method for cancer detection. 

LUNX (lung-specific X protein) is a lung-specific gene that is highly expressed in the NSCLC type of lung cancer. Experimental evidence suggests that LUNX may be considered as a diagnostic biomarker for lung cancer and be able to determine micro-metastases in lymph nodes of NSCLC patients and peripheral blood [15, 16]. Sequential analysis shows that LUNX may play a role in intrinsic immunity [17]. The exact morphologic function of this gene is unknown, but it plays a role in inflammatory responses to upper respiratory tract stimulation. Determination of the expression level of this marker in lung cancer patients by real-time RT-PCR (qRT-PCR) can therefore be beneficial in its earlier stages.

18s rRNA (18s subunit of ribosomal RNA) is a housekeeping gene which was reviewed in a similar study [18]. By measuring its level of expression through qRT-PCR method, this gene can also be considered as a reference in this study. Few studies have been conducted on the expression of the mentioned markers in peripheral blood so far [18]. Therefore, the main objectives of this study were to determine the levels of LUNX mRNA and CEA mRNA in peripheral bloods of NSCLC cancer patients through qRT-PCR and to study their sensitivity and specificity individually and in combined form. The other goal was to offer an applicable method to increase the sensitivity and specificity of these markers to enable their use in the early diagnosis of lung cancer before its metastasis. 

## MATERIALS AND METHODS

Two groups of affected and healthy individuals were involved in this study. The first group consisted of stage I to III lung cancer patients (approved by pathologic tests) visiting Masih Daneshvari Hospital (A center of lung disease therapy), Tehran, Iran. The exclusion criteria for this group were a history of medical or surgical treatments. The healthy group consisted of the individuals who had visited the same hospital in the same duration of time with a normal bronchoscopic or pathologic finding. This group was matched with the patients in terms of age and gender. 

***Blood sampling. ***A written consent was obtained from each participant and a questionnaire covering personal data and disease status was completed by researcher. Then, 12-ml peripheral blood sample was taken by venipuncture. Considering the possibility of epithelial cell contamination, the first 2 ml of the sample was discarded, but the remaining 10 ml was divided into two 8-ml and 2-ml parts. The 8 ml was collected in an EDTA-treated falcon, and the 2 ml was allowed to clot for serum extraction in another tube. Eventually, both tubes were packed on ice and transferred to the laboratory.

***Red blood cell lysis. ***RBC lysis buffer (0.01M Tris-HCl pH 7.4, 320 mM sucrose, 5 mM MgCl_2_, 1% Triton X-100) added to the sample. The resultant solution was vortexed for 30 minutes to allow complete RBC lysis and centrifuged at 4,000 ×g for 20 minutes. Finally, the resultant sediment (including white blood cells and possible cancerous cells) entered the RNA extraction stage.

***RNA extraction. ***This stage was performed with the RNeasy Midi Kit (Qiagen, Germany). The quality and quantity of the extracted RNA was controlled by the NanoDrop spectrophotometer (Bio-TeK, USA). Regarding the concentration of the extracted RNA and maximum capacity of the cDNA synthesis kit, the extracted RNA was immediately applied for cDNA synthesis.

***Reverse transcription. ***The resultant RNA (3 µg) was converted to cDNA in three separate vials (each containing 1 µg) by the Viva 2-steps RT-PCR Kit (Vivantis Technologies, Malaysia) and kept at -80°C. The cDNA quantity was measured by a nanodrop spectrophotometer (Bio-TeK, USA), and the quality of the cDNA was approved for qRT-PCR detecting 18s rRNA expression. The final test was performed separately on each vial sample.

***Primers. ***The specific primers of each marker were designed with AlleleID 7 software (Premier Biosoft, Palo Alto, USA) and ordered for synthesis. Table 1 shows the parameters and amounts used in the final qRT-PCR. 

***Quantitative qRT-PCR. ***In order to investigate the presence of LUNX mRNA and CEA mRNA, the cDNA vials were tested through the qRT-PCR technique using the HotTaq Eva Green qPCR Mix Kit (Sinaclon, Iran) in a CFX96 qRT-PCR detection system (Bio-Rad, USA). The reaction components of qRT-PCR were: a) 0.1 µg of template, b) 4 µl of Master mix, c) the most appropriate primer concen-tration found in the initial set up tests (Table 1), and d) deionized distilled water up to a final volume of 20 µl. Positive and negative controls were simultaneously carried out for quality control of the method and detection of possible cases of contamination.

**Table 1:** Properties of primers used in the real-time RT-PCR reaction. Each gene number, sequence, size, and amount of primer used has been specified separately

**Property**	**CEA**	**LunX**	**18s rRNA**
NCBI accession number	M29540	NM_016583.3	X03205
Forward primer	accctggatgtcctctatgg	CCACCGTCTCTATGTCACCA	gtaacccgttgaaccccatt
primer length	20	20	20
amount of use	15 picomol	10 picomol	10 picomol
Reverse primer	caggcataggtcccgttatta	GCCAAGTCCATCAAGCAGA	ccatccaatcggtagtagcg
primer length	21	19	20
amount of use	10 picomol	10 picomol	10 picomol
Amplicon length	174	211	152
Optimized annealing temperature	61.2°C	61.4°C	53.5℃

CEA, carcinoembryonic antigen

***Statistical methods. ***The sample size was calculated based on the positivity of the markers in the two groups, 5% type I and 20% type II errors. The estimate was 25 diseased and 25 healthy individuals. However, the sample size was increased to 30 in order to increase the statistical power. Statistical analysis was carried out using SPSS 10. The mean values of the two groups were compared with *t*-tests, and the marker positivity was compared with the ‘two-sample binomial’ comparison test. A *P*≤0.05 was considered significant.

## RESULTS

There were 23 males and 7 females in the case/ diseased group, and 24 males and 6 females in the control/healthy group. A comparison of mean age in the cases (mean = 48.4 ± 9.25) and controls (mean = 44.3 ± 8.58) did not show any significant difference (*P* = 0.73). The comparison between females (*P* = 0.46) and males (*P* = 0.082) indicated no significant difference between these subgroups either. 

***Measuring the expression of the study reference gene (18s rRNA). ***The level of reference gene (18s r RNA) expression, which was approximately determined from the cycle threshold (ct) value, was measured for each sample. The mean value of this index between the cases (17.93) and controls (15.76) showed no significant difference between the two groups (*P* = 0.063), approving the selection of this gene as a reference.

***Genetic expression analyses of markers (CEA mRNA and LUNX mRNA). ***The CEA mRNA marker was positive in 24 out of 30 patients, indicating a sensitivity of 80%. It was also positive in 11 out of 30 healthy individuals, showing a 36.6% false-positive rate. Statistically, the difference between the two groups was significant (*P*<0.001). The LUNX mRNA was positive in 21 out of 30 patients, showing a sensitivity of 70%. LUNX mRNA marker expression in patients indicated 100% specificity. For examination of whether increasing the number of vials raised the marker sensitivity or not, the positivity rate of each vial for each marker (vials 1, 2, and 3, respectively) were compared with the overall positivity rate of that marker. There were significant differences in vials 2 and 3 regarding the CEA mRNA marker, but none was observed in vial 1. The comparison of the LUNX mRNA marker vials also showed significant differences in vials 1 and 3 but not in vial 2 (Table 2).

**Table 2:** The comparison of positivity in lung cancer patients’ vials, separately for each vial number, and each marker’s sensitivity using the two-sample binomial test

**Vial **	**CEA mRNA**				**Lunx mRNA**			
	**Positive rate** **(%)**	**Sensitivity (%)**	***P*** **value**		**Positive rate** **(%)**	**Sensitivity (%)**	***P*** **Value**	
1	73	80	0.4		36	70	0.02
2	60		0.017		53		0.73
3	60		0.017		46		0.04

CEA, carcinoembryonic antigen

**Table 3:** The simultaneous positive marker status in lung cancer patients. The table shows the numbers of individuals in each subgroup

**Patient group**		**CEA mRNA**	
		**positive**	**negative**
LUNX mRNA	positive	17	4
	negative	7	2

***Difference in marker expression in the two groups. ***The ΔΔCt method was used to examine the difference in marker expression between patients and healthy individuals with positive markers. The computed ΔΔCt for CEA mRNA was -4.95, showing that the relative expression of this marker was on average 24.50 times that found in healthy individuals. Means of ΔCt were 17.27 ± 6.82 in cases and 22.22 ± 3.92 incontrols for CEA mRNA. A comparison of ΔCt level in CEA mRNA showed a significant difference between the two groups (*P*<0.001). In patients, means of ΔCt for LUNX mRNA was 16.57 ± 4.73. Expression of this gene was not observed in healthy individuals; therefore, this gene will specifically express in patients with lung cancer. 

***Results of simultaneous analysis of marker. ***Totally, 28 patients had at least one positive marker, which showed a rate of 93.3%. This sensitivity was compared with the individual sensitivity of each marker. The difference observed for CEA mRNA was not significant (*P* = 0.134), whereas for LUNX mRNA, it was significant (*P* = 0.02) (Table 3).

## DISCUSSION

Lung cancer is the leading cause of death and the most common type of cancer in the world [19]. Similar to other cancers, earlier diagnosis leads to more effective treatment and better prognosis. Among the non-invasive techniques applied during the initial stages of cancer, tumor markers hold a respectable place in the early detection of disease. Among the various types of tumor markers, desirable properties of mRNA allow them a unique position among the other lung cancer diagnostic tests; hence, the research team interested in its choice of research. One of these properties is their ability to be detected even in very minute quantities through the qRT-PCR technique, which is a highly sensitive and specific technique. Here, we attempted to determine the sensitivity and specificity of CEA mRNA and LUNX mRNA, as specific lung cancer tumor markers, in peripheral blood. So far, studies on these two biomarkers have mostly concentrated on increasing their diagnostic accuracy of detecting metastases. Others have investigated their simultaneous presence in peripheral blood [20, 21]. 

Earlier studies have shown a significant rise in mRNA sensitivity associated with increased numbers of sampling [22, 23]. Hence, the relatively low sensitivity in single tests compared to multiple tests could be attributed to the lower chance of detecting the marker. In other words, marker expression in blood samples is extremely low because of being in the initial stages of disease and the scanty presence of cancerous cells in blood. Therefore, statistically speaking, the possibility of finding the marker is increased upon test repetition [24]. Thus, instead of repeated sampling, the frequency of testing each sample increased from one to three times. This procedure was used in a similar study conducted on other types of mRNA in lung cancer, and had resulted in a significantly increased marker sensitivity [24]. As shown in Table 2, this procedure resulted in similar findings. However, since most studies in this field have been conducted during the final stages of disease, the marker sensitivities reported are slightly higher than ours (80% for CEA mRNA) [7]. Iwao [15] have shown that LUNX mRNA expression is enhanced in 26 (84%) out of 31 NSCLC tumors. Wallace [16] have found that this marker was in the highest sensitivity of (15 out of 27 [56%]) NSCLC tumors, which is similar to our study (70% for *LUNX* mRNA).

The correlation between the two markers of this study is in unison with that between CEA mRNA and CK19 mRNA in Bates study (either CYFRA 21.1 or CEA was elevated in 70% of patients) [25]. This finding is however not similar to the study conducted in 1995 [26]. 

One study investigated the expression of LUNX mRNA in lung cancer patients and found 14 out of 24 positive instances (58%) [14]; however, this rate was increased to 70% in the current study. In other study, the Lunx mRNA-positive rate was determined to be 44.2% for patients with NSCLC, which was less than this literature [27].

CEA mRNA marker expression in afflicted individuals was many times more than healthy ones. Specificity of this marker for diagnosing and screening of lung cancer was 60% and false positive was 36.6%. Because the expression of this marker in patients is more than normal people; nonetheless, it could be used as a diagnostic marker. LUNX mRNA marker expression in patients indicated 100% specificity.

LUNX mRNA and CEA mRNA markers in 17 out of 30 patients were positive and 2 out of 30 patients were negative simultaneously, indicating that the two markers had suitable sensitivity and specificity for lung cancer screening.

 This study suggests that the combination of LUNX mRNA and CEA mRNA markers of peripheral blood may be considered as a useful tool to detect non-small cell lung cancer by qRT-PCR.

In conclusion, it was detected that the level of LUNX and CEA mRNA in peripheral blood might be suggested as convenient markers of non-invasive lung cancer with a desirable sensitivity, providing our findings will be approved by further extensive studies on larger sample sizes.
